# Residual Effect of Microbial-Inoculated Biochar with Nitrogen on Rice Growth and Salinity Reduction in Paddy Soil

**DOI:** 10.3390/plants13192804

**Published:** 2024-10-06

**Authors:** Hafiz Muhammad Mazhar Abbas, Ummah Rais, Haider Sultan, Ashar Tahir, Saraj Bahadur, Asad Shah, Asim Iqbal, Yusheng Li, Mohammad Nauman Khan, Lixiao Nie

**Affiliations:** 1 School of Breeding and Multiplication (Sanya Institute of Breeding and Multiplication), Hainan University, Sanya 572000, China; mazharabbas@hainanu.edu.cn (H.M.M.A.); sultanhaider@hainanu.edu.cn (H.S.); 184459@hainanu.edu.cn (A.S.); yushengli@hainanu.edu.cn (Y.L.); 2 Department of Zoology, The Islamiyah University of Bahawalpur, Bahawalpur 63100, Pakistan; ummahasghar2301@gmail.com; 3 School of Tropical Agriculture and Forestry, Hainan University, Haikou 570228, China; drashartahir@hainanu.edu.cn; 4 College of Forestry, Hainan University, Haikou 570228, China; saraj@hainanu.edu.cn; 5 Department of Agronomy, University of Agriculture Faisalabad, Faisalabad 37000, Pakistan; aximiqbal60@gmail.com

**Keywords:** residual effects, bacterial biochar, fungal biochar, saline water, root cross-section

## Abstract

Increasing soil and water salinity threatens global agriculture, particularly affecting rice. This study investigated the residual effects of microbial biochar and nitrogen fertilizer in mitigating salt stress in paddy soil and regulating the biochemical characteristics of rice plants. Two rice varieties, Shuang Liang You 138 (SLY138), a salt-tolerant, and Jing Liang You 534 (JLY534), a salt-sensitive, were grown under 0.4 ds/m EC (S0) and 6.84 ds/m EC (S1) in a glass house under controlled conditions. Three types of biochar—rice straw biochar (BC), fungal biochar (BF), and bacterial biochar (BB)—were applied alongside two nitrogen (N) fertilizer rates (60 kg ha^−1^ and 120 kg ha^−1^) in a previous study. The required salinity levels were maintained in respective pots through the application of saline irrigation water. Results showed that residual effects of microbial biochars (BF and BB) had higher salt mitigation efficiency than sole BC. The combination of BB and N fertilizer (BB + N120) significantly decreased soil pH by 23.45% and Na^+^ levels by 46.85%, creating a more conducive environment for rice growth by enhancing beneficial microbial abundance and decreasing pathogenic fungi in saline soil. Microbial biochars (BF and BB) positively improved soil properties (physicochemical) and biochemical and physiological properties of plants, ultimately rice growth. SLY138 significantly had a less severe response to salt stress compared to JLY534. The mitigation effects of BB + N120 kg ha^−1^ were particularly favorable for SLY138. In summary, the combined residual effect of BF and BB with N120 kg ha^−1^, especially bacterial biochar (BB), played a positive role in alleviating salt stress on rice growth, suggesting its potential utility for enhancing rice yield in paddy fields.

## 1. Introduction

Salinization of soil or irrigation water is a major environmental concern that threatens agricultural production, food security, and sustainable development [1,2,3]. Inefficient saline water management leads to soil and water salinization, adversely affecting human health, natural resources (plants, animals, aquatic ecosystems), and agricultural and infrastructural sustainability [4]. Estimates indicate a global prevalence of saline soils at roughly 11.3 million hectares, with an annual expansion of 1.0–1.5 million hectares [5,6]. Salinization is projected to impact nearly half of all agricultural land by 2050 [7], highlighting the urgency for developing ecological restoration and cultivation strategies in saline environments. The complex and persistent nature of salinization in irrigated soils necessitates a multi-faceted approach for successful remediation.

Salinity has significant negative effects on the growth, physiological attributes, and biochemical processes of plants by disturbing the availability of soil nutrients [8]. Similarly, Jameel et al. [9] also reported that salinity adversely affected shoot and root morphological attributes and photosynthetic as well as biochemical properties of the brinjal plant. Salinity also has an indirect effect on plant growth as it can negatively affect soil properties, availability of nutrients, soil water relations, and ultimately soil-plant relations [8,10].

Biochar-based fertilizers increased rice productivity by 15 to 30%, with nitrogen use efficiency (NUE) around 80% [11]. This finding supports the use of biochar in agricultural fields as a soil conditioner to improve the effectiveness of conventional nitrogen fertilizers. However, according to Mehdizadeh [12] and Wang [13], despite its extensive use, the application of biochar in saline soils presents a notable challenge due to its slow decomposition rate and delayed nutrient release. The modification of biochar with effective microorganisms has demonstrated its efficacy as a microbial carrier, attributed to its porous structure and microbial affinity [14]. Studies have observed significant increases in NUE. For instance, Steiner [15] reported an 18% increase in NUE with NPK fertilizer combined with biochar compared to plots receiving only NPK.

Co-application of biochar and effective microorganisms presents an eco-friendly strategy to enhance plant growth and remediate saline soil [16], offering new avenues for managing coastal saline-alkali environments [17]. The synergistic effect of biochar and microbes (Chlorella) increased plant growth and ultimately yield by improving the physicochemical properties and nutrient status of saline soil [18]. Similarly, microbial and biochar interactions synergistically increased plant biomass both aboveground and belowground by improving soil properties through enhanced nutrient availability and improved soil physical properties [19]. Biochar application has primarily demonstrated positive effects on crop yield in nutrient-depleted soils, likely due to enhanced nutrient availability and retention [20]. Combining biochar with nitrogen fertilizer can promote sustainable crop production by reducing nitrogen losses, improving nitrogen use efficiency (NUE), and enhancing soil properties [21].

The positive influence of biochar, particularly when combined with nitrogen fertilizer, on agricultural productivity is well documented [21,22]. Moreover, Li et al. [23] and Lu et al. [24] reported that when N is applied with biochar, it enhances the efficiency of biochar to remediate deteriorated soil, as N can affect the biological attributes of soil. Studies have shown that long-term application of biochar, along with nitrogen fertilizer, enhances soil physical and chemical properties. It was reported that the combined application of biochar (60 t ha^−1^) and N (360 kg ha^−1^) improved soil physicochemical properties, nutrient uptake, and physiological properties of plants and ultimately enhanced plant growth significantly than the sole application of biochar and nitrogen [25]. Moreover, when biochar and nitrogen are applied together, biochar enhances nitrogen availability, and higher availability of nitrogen improves the physiological attributes of plants and plant growth [26]. These improvements in the soil environment contribute to better plant morphology and ultimately lead to increased crop yields [27]. However, according to our information, a knowledge gap exists regarding the long-term combined effects of microbial biochar and nitrogen fertilizer on agricultural productivity.

Hence, we hypothesized the following: (1) microbial biochar and N may synergistically mitigate the negative effects of saline water on plants by improving soil properties and consequently enhancing plant growth under these stressful conditions. (2) N fertilizer may assist significantly microbial biochar to mitigate salinity stress in the paddy field to grow rice crops. Previous studies on the combined application of biochar and nitrogen fertilizers have primarily focused on short-term observations following a single season. This research addresses this gap by investigating the long-term effects (residual effects) of microbial-inoculated biochar combined with nitrogen fertilizer on rice growth and soil nutrient conditions within a Rice-Fallow-Rice system. The study specifically aimed to assess how microbial-inoculated biochar application influences crop yield and soil nutrients over time and to evaluate whether the beneficial effects of microbial biochar and nitrogen fertilizers on rice growth and soil health in saline soil persist over extended cultivation cycles within a Rice-Fallow-Rice system.

## 2. Results

### 2.1. Chemical Properties of Post-Harvest Soil

**Figure 1:**
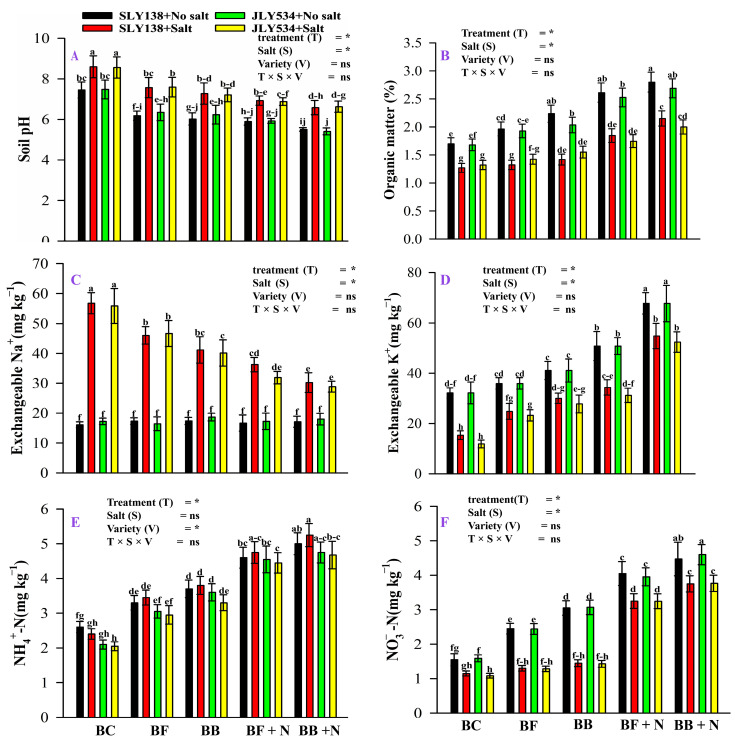
The residual effect of biochar, microbial-inoculated biochar, and N on soil physicochemical properties for growing conditions of both varieties (**A**) soil pH, (**B**) soil organic matter, (**C**) exchangeable Na^+^, (**D**) Exchangeable K^+^, (**E**) NH_4_^+^-N accumulation, and (**F**) NO_3_^−^-N accumulation. BC (simple biochar); BF (fungal biochar); BB (bacterial biochar); BF + N (fungal biochar and nitrogen); BB + N (bacterial biochar and nitrogen). The means that have the same letter do not differ substantially at *p* > 0.05 for a parameter, significant * (*p* ≤ 0.05), non-significant (ns; *p* > 0.05). T (Treatment), S (Salt), V (Variety).

The effect of salinity, biochar, microbial biochar, and nitrogen on the chemical properties of soil is shown in (Figure 1). The synergistic effect of bacterial biochar and nitrogen (BB + N120) decreased the soil pH by 23.45% compared to rice straw biochar (BC) under saline conditions (Figure 1A). Salt stress and rice straw biochar increased soil pH; however, microbial-inoculated biochar (BF and BB) and nitrogen decreased soil pH. Compared to simple biochar (BC), BB + N120 increased OM in soil by 70% under stress conditions. Salinity decreased OM in soil by 25% in BC treatment (Figure 1B). As expected, Na^+^ increased in saline conditions; however, the synergistic effect of bacterial biochar (BB) and N decreased Na^+^ by 46% than BC treatment (Figure 1C). Usually, salinity decreases K^+^ content in the soil, while organic amendment increases K^+^ content in the soil. In our study, salinity decreased K^+^ content by 52.34% in BC treatment and BB + N120 amendment increased exchangeable K^+^ by 258% compared to BC in saline conditions (Figure 1D). Among treatments, BB + N120 enhances NH_4_^+^-N in soil by 127% under saline conditions. Salinity decreased, NO_3_^−^-N accumulation in soil by 25% in BC treatment (Figure 1E). Compared with control, BB + N120 increased NO_3_^−^-N accumulation in soil by 226% under stressed conditions (Figure 1F).

### 2.2. Synergistic Effect of Microbial Biochar and Nitrogen Fertilizer Modulated K^+^/Na^+^ Balance in Rice Leaves under Saline Conditions

**Figure 2:**
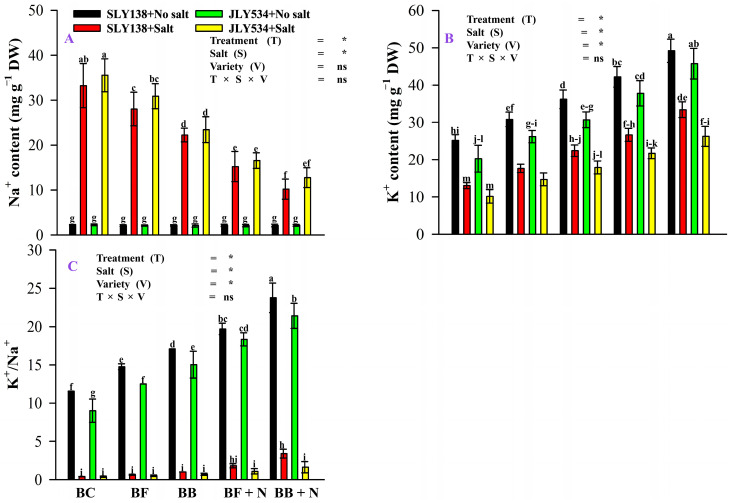
The residual effect of different biochars and N on Na^+^, K^+^, and K^+^/Na^+^ in rice leaves of both varieties; (**A**) Na^+^ content; (**B**) K^+^ content; (**C**) K^+^/Na^+^. BC (simple biochar); BF (fungal biochar); BB (bacterial biochar); BF + N (fungal biochar and nitrogen); BB + N (bacterial biochar and nitrogen). The means that have the same letter do not differ substantially at *p* > 0.05 for a parameter, significant * (*p* ≤ 0.05), non-significant (ns; *p* > 0.05). T (Treatment), S (Salt), V (Variety).

As expected, rice leaves grown under saline conditions exhibited increased Na^+^ content compared to those grown in non-saline conditions (Figure 2A). Interestingly, under saline conditions, rice leaves from the BB + N120 treatment displayed a significant 69% and 64% reduction for SLY138 and JLY534, respectively, in Na^+^ content compared to plants receiving only BC treatment (Figure 2A). All treatments significantly impacted rice leaf K^+^ content under both saline and non-saline conditions (Figure 2B). Consistent with previous observations, saline conditions led to a decrease in rice leaf K^+^ content compared to non-saline conditions. Under saline stress, rice leaves from the BB + N treatment remarkably maintained 156% and 159% greater K^+^ content for SLY138 and JLY534 cultivars, respectively, compared to those from the BC treatment. The substantial alterations in Na^+^ and K^+^ content resulted in a superior K^+^/Na^+^ ratio for the BB + N120 treatment under saline conditions (Figure 2C). Notably, the BB + N120 treatment exhibited 757% and 328% higher K^+^/Na^+^ ratios in the SLY138 cultivar and JLY534, respectively, compared to the BC treatment.

### 2.3. Microbial Biochar and Nitrogen Fertilizer Reduced the ROS Damaging Effects in Rice Leaves and Increased SOD, POD, and CAT Activities in Rice Plants

**Figure 3:**
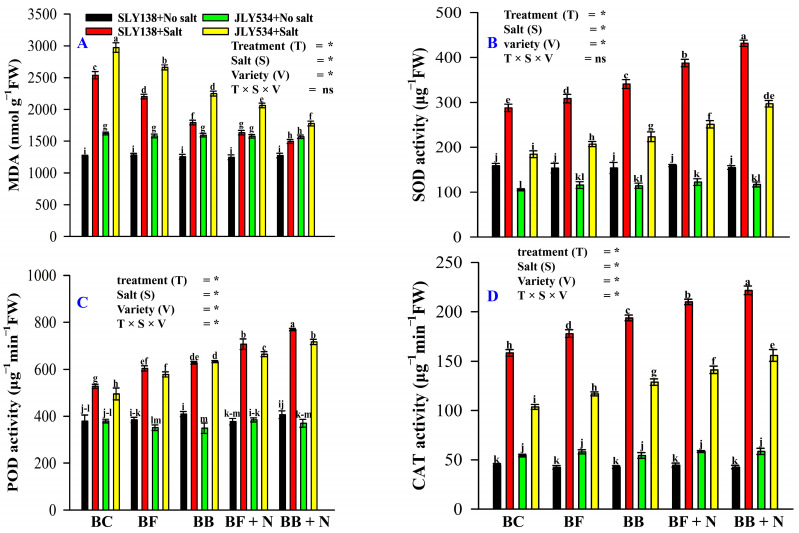
The residual effect of biochars and N on MDA, SOD, POD, and CAT in rice leaves of both varieties: (**A**) MDA activities, (**B**) SOD activities, (**C**) POD activities, and (**D**) CAT activities. BC (simple biochar); BF (fungal biochar); BB (bacterial biochar); BF + N (fungal biochar and nitrogen); BB + N (bacterial biochar and nitrogen). The means that have the same letter do not differ substantially at *p* > 0.05 for a parameter, significant * (*p* ≤ 0.05), non-significant (ns; *p* > 0.05). T (Treatment), S (Salt), V (Variety).

No significant differences were observed in malondialdehyde (MDA) content or the activities of antioxidant enzymes among treatments for rice leaves grown under non-saline conditions for both varieties (Figure 3). Salinity markedly elevated MDA levels in leaves, signifying salt-induced plant injury (Figure 3A). Rice leaves from the BC treatment exhibited a 41% and 40% increase in MDA content for SLY138 and JLY534, respectively, compared to those from the BB + N120 treatment, indicating greater membrane damage under saline conditions. Rice leaves from the BB + N120 treatment displayed significantly reduced malondialdehyde (MDA) content (41% and 40% for SLY138 and JLY534, respectively) compared to the BC treatment under saline conditions. This suggests that BB + N120 effectively mitigated ROS production, alleviating membrane damage in rice plants exposed to salt stress. The BB + N120 treatment significantly enhanced the activities of antioxidant enzymes, superoxide dismutase (SOD), peroxidase (POD), and catalase (CAT), by 50%, 45%, and 39%, respectively, for the SLY138 cultivar and 61%, 44%, and 50% for JLY534 under saline conditions compared to the BC treatment (Figure 3B-D). Consistent with previous observations under non-saline conditions, no significant differences in the activities of superoxide dismutase (SOD), peroxidase (POD), and catalase (CAT) were detected among treatments for both rice varieties.

### 2.4. Microbial Biochar and Nitrogen Fertilizer Maintain RWC and MSI in Rice Plants under Salinity Stress

**Figure 4:**
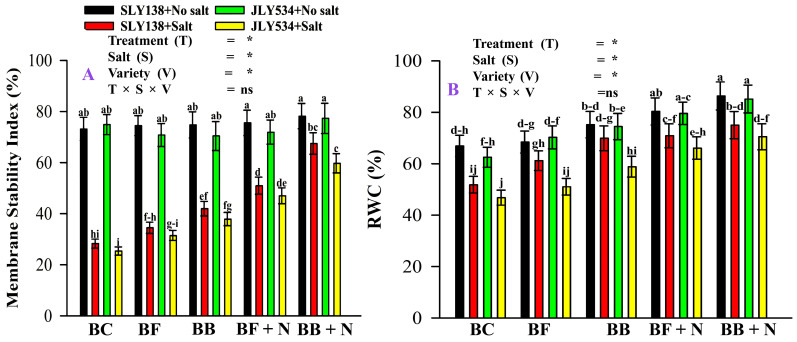
The residual effect of biochars and N on RWC and MSI in rice plants of both varieties, (**A**) RWC and (**B**) MSI. BC (simple biochar); BF (fungal biochar); BB (bacterial biochar); BF + N (fungal biochar and nitrogen); BB + N (bacterial biochar and nitrogen). The means that have the same letter do not differ substantially at *p* > 0.05 for a parameter, significant * (*p* ≤ 0.05), non-significant (ns; *p* > 0.05). T (Treatment), S (Salt), V (Variety).

While no significant differences in relative water content (RWC %) were observed among treatments for either rice variety under non-saline conditions, the membrane stability index (MSI%) was significantly affected (Figure 4). Under saline conditions, all treatments significantly affect the RWC (%) of both varieties. Overall, salt stress reduced relative water content by 22% and 25% for SLY138 and JLY534, respectively, in BC treatment. The BB + N120 treatment significantly enhanced the RWC by 45% and 50% for SLY138 and JLY534, respectively, compared to the BC treatment under saline conditions (Figure 4A). Also, BB + N120 treatment improved the membrane stability index by 138% and 135% for SLY138 and JLY534, respectively, under saline conditions (Figure 4B).

### 2.5. Microbial Biochar and Nitrogen Fertilizer Mitigate Salinity Stress and Enhance Photosystem II Function in Rice Plants

**Figure 5:**
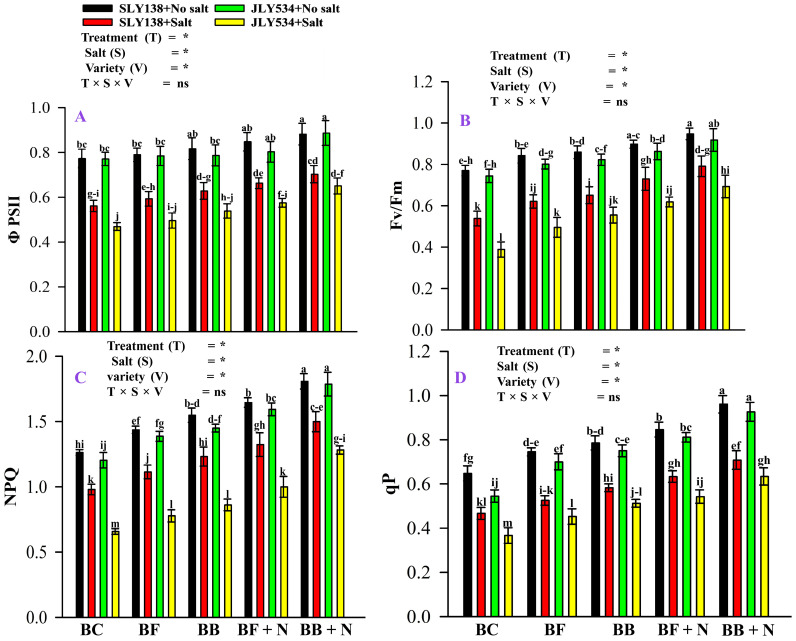
The residual effect of Biochars and N on (ΦPSII), (Fv/Fm), (NPQ), and (qP) in rice leaves of both varieties, (**A**) ΦPSII, (**B**) Fv/Fm, (**C**) NPQ, and (**D**) qP. BC (simple biochar); BF (fungal biochar); BB (bacterial biochar); BF + N (fungal biochar and nitrogen); BB + N (bacterial biochar and nitrogen). The means that have the same letter do not differ substantially at *p* > 0.05 for a parameter, significant * (*p* ≤ 0.05), non-significant (ns; *p* > 0.05). T (Treatment), S (Salt), V (Variety).

Microbial-modified biochar, nitrogen fertilizer, and salinity stress significantly affect (*p* < 0.05) the photosynthesis of rice plants (Figure 5). Salinity stress significantly affects the functions of photosystem II of rice plants. Under saline conditions, all treatments significantly affect photosystem II of both varieties. BB + N120 treatment enhanced photon yield of Photosystem II (ΦPSII), chlorophyll fluorescence (Fv/Fm), non-photochemical quenching (NPQ), and photochemical quenching coefficient (qP) by 25%, 47%, 53%, and 52%, respectively, for SLY138 and 39%, 78%, 95%, and 73%, respectively, for JLY534 compared to BC treatment under saline conditions.

### 2.6. Effect of Microbial Biochar and Nitrogen Fertilizer on Roots Anatomical Properties under Saline Stress

**Figure 6:**
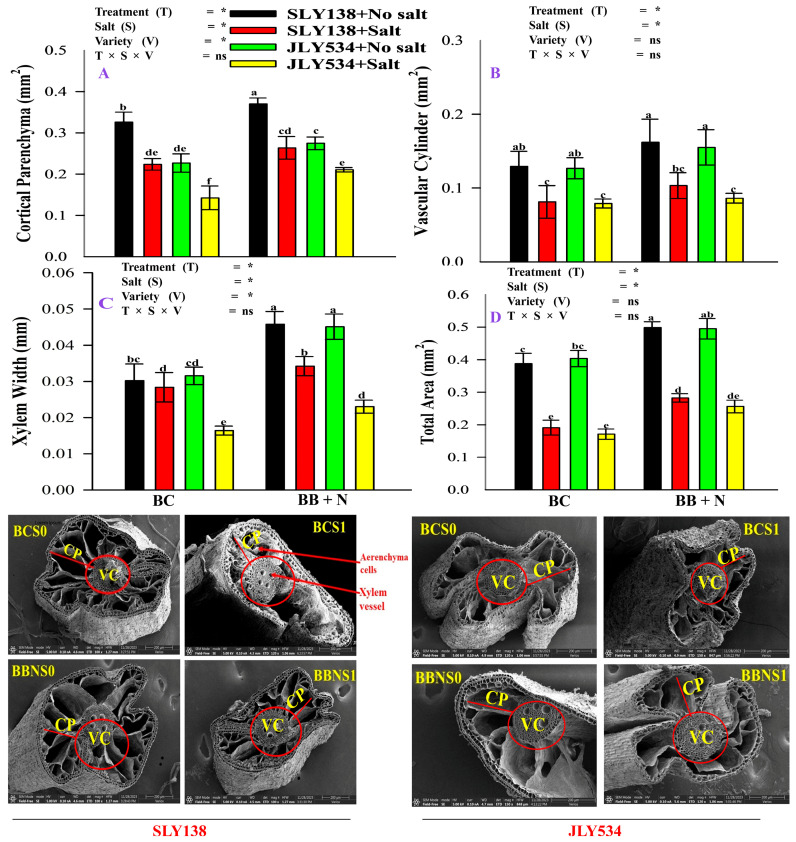
The residual effect of Biochars and N on CP, VC, xylem width, and total area of root cross-section in rice roots of both varieties, (**A**) CP (**B**) VC (**C**) Xylem width (**D**) Total area of root cross-section: SLY138 (V1); JLY534 (V2): CP (Cortical parenchyma); VC (Vascular cylinder); BC (simple biochar); BB + N (bacterial biochar and nitrogen); BCS0—rice straw biochar applied into the soil with no salt; BCS1—rice straw biochar applied into the soil irrigated with saline water; BBNS0—bacterial biochar and N applied into the soil with no salt; BBNS1—bacterial biochar and N applied into the soil irrigated with saline water; N-nitrogen applied at the rate of 120 kg ha^−1^. The means that have the same letter do not differ substantially at *p* > 0.05 for a parameter, significant * (*p* ≤ 0.05), non-significant (ns; *p* > 0.05). T (Treatment), S (Salt), V (Variety).

Salt stress resulted in reductions in the total root area, vascular cylinder area, cortex area, and xylem width in rice plants (Figure 6). Under saline conditions, the BB + N120 treatment significantly improved rice root anatomy, as evidenced by increases in cortical parenchyma (CP) area by 18% and 48% for SLY138 and JLY534, respectively; vascular cylinder (VC) area by 27% and 9% for SLY138 and JLY534, xylem width by 21% and 40% for SLY138 and JLY534; and total root area by 48% and 49% for SLY138 and JLY534, respectively (Figure 6).

### 2.7. Ultrastructure Traits of Rice Leaf

**Figure 7:**
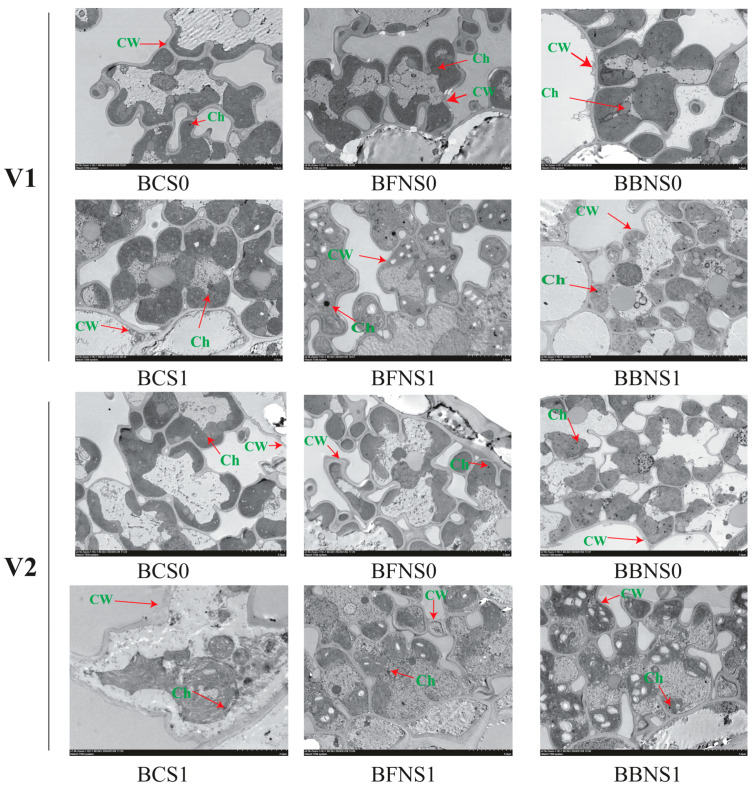
The ultrastructure of leaf cells in rice seedlings for SLY138 (V1) and JLY534 (V2) (B). S0—0% NaCl; S1—0.4% NaCl; BC—rice straw biochar applied into the soil; BF—Fungal biochar produced applied into the soil; BB—biochar produced applied into the soil; N—nitrogen applied at the rate of 120 kgha^−1^. CW—cell wall; Ch—chloroplast.

Cell elongation and cell division are essential processes for plant growth and development. Transmission electron microscopy (TEM) revealed that salt stress triggered chloroplast deformations of SLY138, including shape distortions and unclear membrane systems (Figure 7). Rice leaves subjected to the combined treatment of bacterial biochar (BB) and N120 fertilizer exhibited improved cell structure and morphology under saline conditions. Notably, the BB + N120 treatment resulted in well-defined and organized cells compared to the BC treatment alone. TEM analysis revealed contrasting responses to salt stress between rice varieties JLY534 and SLY138 (Figure 7). Under salt stress, JLY534 chloroplasts exhibited severe separation from the cell wall and disrupted membrane systems. SLY138, however, displayed minimal chloroplast disruption. The application of bacterial biochar, particularly with nitrogen fertilizer (BB + N120), significantly improved packed grana in both varieties, likely due to the alleviated salt stress in the soil following biochar and N application.

### 2.8. Agronomic Parameters and SPAD Values of Rice

Rice variety, salinity stress level, biochar type, and nitrogen application rate significantly affected the agronomic parameters of rice plants (Table 1 and Table 2). Salinity stress significantly reduced plant height, tiller number, and both fresh and dry weight in rice plants of both varieties. Notably, JLY534 exhibited a greater decline in these parameters compared to SLY138, suggesting a difference in salt tolerance between the two cultivars. Further morphological performance of SLY138 under saline conditions with biochar application (BC). Compared to the control, BC treatment resulted in a 25% increase in plant height, a 17% increase in plant fresh weight, a remarkable 100% increase in dry weight, and a 5% increase in the number of tillers for SLY138. The observed significant varietal differences in salt tolerance extended to chlorophyll content (SPAD), with SLY138 exhibiting a 6.8% higher SPAD value under saline conditions. Furthermore, both fungal biochar (BF) and bacterial biochar (BB) applications significantly improved these morphological parameters in both rice varieties. This positive effect was further amplified by increasing nitrogen application rates. These findings suggest that biochar amendment, particularly in combination with nitrogen fertilizer, presents a promising strategy to mitigate the detrimental effects of salinity stress on rice growth, especially for salt-sensitive cultivars.

## 3. Discussion

In our study, NaCl increased soil pH and Na^+^ content in soil and decreased OM, K^+^, NH_4_^+^-N, and NO_3_^−^-N, while biochar application increased soil pH and biochar-based microbial agents with N decreased soil pH, Na^+^ content, increased OM, and available nutrients (NH_4_^+^-N and NO_3_^−^-N) in saline soil (Figure 1). Similarly, many researchers reported that biochar and N fertilizer improved soil chemical properties under saline conditions [28,29,30,31]. Following an increase in soil pH due to salt and biochar, microbial-modified biochar (BF and BB) and N decreased soil pH (Figure 1A). Saline water irrigation can elevate soil pH due to reduction reactions triggered by flooding [32] as these reactions consume H^+^ ions, leading to a more alkaline soil environment. Consistent with previous findings on biochar and microbial-modified biochar [33,34], our experiment observed similar changes in soil pH during incubation. On the other hand, nitrification is an acidifying process that can lead to a reduction in pH levels [35,36]. Moreover, the proliferation of acid-producing soil microorganisms in biochar-amended soil may also lower soil pH levels [37]. It was shown that bacterial biochar (BB) combined with N reduced the soil pH even under saline conditions. Many studies showed that N-containing fertilizers can lead to a slight decrease in soil pH due to proton release during soil nitrification [38,39].

Saline conditions have negative effects and reduced OM in our soil (Figure 1B). Similar to our findings, many studies reported that inhibition of OM accumulation in saline environments is associated with reduced primary productivity and lower-quality organic matter input [40,41]. It might be due to the negative impact of high Na^+^ concentrations on soil aggregate formation and the subsequent increased susceptibility of organic matter to loss [42]. Microbial decomposition is considered the primary mechanism for biochar degradation in soil. This process plays a key role in fueling the activity of inoculated and activated native microbes within the soil environment. As biochar decomposes, it provides a valuable energy source for these microbes, potentially stimulating their growth and activity. Similarly, Lian [43] also reported an increase in organic matter in soil amended with microbial-inoculated biochar. Also, nitrogen addition can enhance soil organic matter (SOM) by both slowing the decomposition of biochar and native SOM and this effect is likely mediated by a shift in the soil microbial community and the resulting changes in biogeochemical cycling [24]. In our study, soil treated with BB + N120 has less Na^+^ compared to BC-treated soil under saline conditions. The limited adsorption capacity of biochar for salt ions may contribute to this phenomenon [44]. Similarly, many other studies also reported that it might be attributed to the high surface area and strong adsorption capacity of the biochar [18,45]. Another possibility reported by Zhang et al. [41] is that inoculated and activated by these native microbes may help biochar to decrease Na^+^ in soil by bioaccumulation. When external salinity increases, some halophilic microbes can absorb salt ions to increase cell osmotic pressure, sometimes exporting sodium ions.

Nitrogen (N) is a nutrient that plays a key role in plant growth and development, absorbed by plant roots in the forms of NH_4_^+^-N and NO_3_^−^-N [46,47]. Biochar addition, with or without added microbes, increased soil organic matter (OM), ammonium (NH_4_^+^-N), nitrate (NO_3_^−^-N), and potassium (K^+^) in saline soil, while, the combined application of biochar, microbes, and nitrogen resulted in greater improvements compared to biochar addition alone. Similarly, many studies showed that biochar alone and combined with microbial agents and N enhanced the nutrient status of saline soil [48,49]. Our study found that adding microbial-inoculated biochar and N increased the availability of inorganic nitrogen (NH_4_^+^ and NO_3_^−^) in the soil more than the BC treatment (Figure 1E–F). Similarly, many studies reported a positive effect of biochar on soil inorganic N [31,50]. Previously, it was reported that the combined application of biochar and effective microorganisms (EM) positively impacts soil inorganic nitrogen content and enzyme activities, likely due to increased microbial activity [17,51], also accelerated soil nitrogen transformations, and enhanced N mineralization [52].

Elevated salinity induces excessive accumulation of sodium (Na^+^) while depleting K^+^ in plant cells, causing ionic toxicity by upsetting the critical Na^+^/K^+^ balance [18,41,53]. In the current study, salt stress significantly increased the accumulation of sodium (Na^+^) in rice plant leaves (Figure 2A). While biochar application presents a potential mitigation strategy in salt-affected soils. Its ability to adsorb Na^+^ on its surface helps alleviate salinity stress [18,45]. Furthermore, Cui et al. [17] demonstrated that combining biochar with effective microorganisms offered greater benefits compared to biochar alone. Application of N also contributes to decreasing Na^+^ content and increasing potassium (K^+^) content. This effect is likely due to the production of osmoregulatory substances that enhance the plant’s ability to adjust to osmotic stress [54]. Our findings align with previous research by Liu et al. [55]. Additionally, Yao et al. [56] suggested that nitrogen fertilization reduces Na^+^ uptake in plants by promoting the synthesis of specific proteins located in the cell membrane. Our study also highlights potential varietal differences in response to salinity stress. While both rice varieties accumulated more sodium (Na^+^) under salt stress, SLY138 exhibited a 6% reduction in Na^+^ content compared to JLY534 in the biochar (BC) treatment. This signifies a greater capacity for SLY138 to limit Na^+^ accumulation, potentially contributing to its enhanced salt tolerance relative to JLY534. Also, Singh et al. [4] and Singh et al. [57] observed that the salt-tolerant variety CSR10 maintained a lower Na^+^/K^+^ ratio in shoots compared to the salt-sensitive MI 48. This suggests restricted sodium translocation from roots to shoots in CSR10. Similarly, Zhang et al. [18] reported that the salt-sensitive variety (Nipponbare) accumulated more Na^+^ and Cl^−^ ions compared to the salt-resistant variety (Jinyuan 85), further supporting the link between efficient sodium exclusion and salt tolerance in rice.

High salinity stress triggers an overproduction of reactive oxygen species (ROS) like MDA [58], thereby inhibiting essential biochemical processes in plants [59,60]. To combat the detrimental effects of reactive oxygen species (ROS) produced under high salinity stress, plants have evolved a sophisticated antioxidant defense system [61]. This self-defense mechanism relies on a coordinated action of various antioxidant enzymes, including superoxide dismutase (SOD), ascorbate peroxidase (APX), peroxidase (POD), and catalase (CAT) [62,63]. These findings support previous research by Manan et al. [64], who observed similar increases in antioxidant enzyme activity in tomato plants under NaCl stress. In our study, microbial-inoculated biochar (BF and BB) with N application synergistically enhanced the antioxidant defense system in both cultivars (Figure 3B–D). Similar to our findings, Tian et al. [54] also reported that nitrogen application mitigates the toxic effects of excessive ROS production caused by salt stress through the increased activity of key antioxidant enzymes like SOD, POD, and CAT. This study suggests the long-term combined application of microbial-modified biochar (BF and BB) and N fertilizer can alleviate the detrimental effects of salt stress on plants. Our study also revealed varietal differences in antioxidant defense mechanisms under saline stress. Compared to the salt-sensitive cultivar (JLY534), the salt-tolerant cultivar (SLY138) exhibited higher antioxidant activity in its leaves (Figure 3). This was evidenced by elevated levels of key antioxidant enzymes, including catalase (Figure 3D), peroxidase (Figure 3C), and superoxide dismutase (Figure 3B), suggesting a more robust antioxidant defense system in SLY138.

Osmotic stress, a key mechanism of salinity-induced damage in plants [65], disrupts cell membrane organization as measured by MSI (membrane stability index). This disruption is considered a primary injury and leads to electrolyte leakage. In the current study, SLY138 has high values of RWC and MSI by 11% and 11.5%, respectively, compared to JLY534 in BC treatments under salt stress (Figure 4A). According to Bangar et al. [66], the ability of the genotype to maintain higher MSI is one of the acquired characteristics under stress conditions. It was reported by Yan et al. [67] that under salinity stress, reduction in RWC is due to the disturbed water content of the plant. Since both MSI and RWC reflect plant responses to osmotic stress, improving soil water availability through biochar application can be beneficial. Our study identified the BB + N120 treatment as the most effective in enhancing both MSI and RWC, suggesting its potential to mitigate the negative effects of salinity stress on plants. Our findings are supported by Lyu et al. [68] that biochar strengthens the defense mechanism of leaves against osmotic stress in amended soil by improving the activity of protective enzymes and the transfer of electrons. According to Ahmad et al. [69], the presence of high porosity in BC could be effective in the retention of water and nutrients in soil. Regarding the alleviation of salt stress, the synergistic effect of BB at 1% and N at 120 kg ha^−1^ was much stronger to restore and improve the leaf ultrastructure than BC (Figure 7). Our results are in line with [18,21].

Salinity stress in higher plants typically reduces the rate of photosynthesis due to limitations in CO_2_ availability, degradation of photosynthetic pigments, and diminished leaf area [70], especially by affecting photosystem II activity and electron transfer [71]. In our study, salinity also disturbs the physiological attributes of both varieties (Figure 5). However, the application of microbial biochar (BB) in combination with nitrogen significantly increased the physiological attributes of rice plants more than the BC treatment alone (Figure 5). Similar to our findings, many other studies also reported that biochar with N enhanced the physiological attributes of plants [21]. Because all the growth attributes of plants depend on efficient nutrient and water uptake from the growing media, coupled with improved nutrient utilization [21].

Irrigation with saline water reduced xylem vessel width, cortical parenchyma (CP), vascular cylinder (VC), and total area of root cross-section (Figure 6). Previously, many studies also reported that salinity stress triggers cellular and tissue-level alterations in plants, leading to reduced root, stem, and leaf cell size [72,73]. Similarly, Kong et al. [74] reported that salinity stress induced anatomical modifications in roots, characterized by an increased exodermis to endodermis ratio and a decrease in central cylinder thickness. However, the application of bacterial biochar combined with N fertilizer mitigates significantly more salinity effects on root anatomy compared to BC treatment (Figure 6). Similar to our study, many other studies also found the same results that biochar and N improved root anatomical features like cortical parenchyma (CP), vascular cylinder (VC), and xylem width [41,75].

Salt stress triggered chloroplast deformations of SLY138, including shape distortions and unclear membrane systems (Figure 7). Sodium salt stress dehydrates and shrinks plant cells, hindering shoot development and elongation, especially in salt-sensitive rice genotypes where visual lesions appear [76,77]. Salinity stress has a negative impact on plant cellular and tissue structure, leading to reduced cell size in roots, stems, and leaves [72]. Nevertheless, under saline conditions, BB + N120 (BBNS1) improved cell structure, cell wall, and chloroplast for both varieties (Figure 7). Supporting our results, [21] also reported that biochar amendment along with nitrogen improved cell structure and shape, resulting in well-defined and established cells.

Rice plants irrigated with saline water show fewer morphological attributes (plant height, number of tillers, and biomass) and SPAD values compared to plants grown under non-saline conditions. Similar to our findings, Temme et al. [78] also reported negative effects of salinity on plant morphology and growth indices. While microbial-inoculation of biochar (BF and BB) combined with a nitrogen application rate of 120 kg ha^−1^ significantly increased plant height (PH), fresh weight (PFW), dry weight (PDW), number of tillers, and SPAD content in saline paddy fields (Table 1). Multiple studies have demonstrated that biochar and N application can significantly enhance agronomic characteristics by improving soil physicochemical properties, increasing nutrient availability, promoting soil microbial activity, and optimizing root morphology and physiology [17,79,80,81,82,83]. Microbial inoculation of biochar (BB) and N likely contributed to the observed morphological improvements through several mechanisms. Firstly, BB application enhanced potassium (K^+^) uptake compared to simple biochar (Figure 1D), potentially leading to greater sodium (Na^+^) removal from the soil exchange complex, particularly in the combined BB + N treatments (Figure 1C). Secondly, these treatments resulted in a significant decrease in soil pH (Figure 1A). Thirdly, BB application increased soil organic matter (SOM) content and available nitrogen (Figure 1B and Figure 1E–F). Finally, biochar and nitrogen application likely improved conditions for soil microorganisms and enzyme activity, potentially enhancing root exudation and optimizing rice root structure, as previously reported by [83].

## 4. Materials and Methods

### 4.1. Experimental Setup

The experiment was conducted in a controlled greenhouse setting at the Hainan University experimental farm. Previously used pots for sowing rice crops filled with 5 kg of soil and treated with different treatments (Table S1) were selected for this experiment. Pre-experimental properties of soil used for both varieties are presented in Tables S3 and S4. Based on morphological characteristics, the most successful treatments were chosen for further analysis and are presented in Table S2. The previous experiment employed two salinity levels achieved by applying NaCl salt solutions at concentrations of 0.4 EC (normal soil—S0) and 6.84 ds/m EC (saline soil—S1). Biochar was incorporated into the soil at a rate of 1% (50 g pot^−1^) by weight before filling the pots. Nitrogen fertilizer was applied in three equal split applications: at sowing, during the seedling stage, and at 40 days after sowing (DAS). The experiment investigated two rice varieties: (1) Shuang Liang You 138 (SLY138), a salt-resistant variety, and (2) Jing Liang You 534 (JLY534), a salt-sensitive variety. To avoid the detrimental effects of salts from the previous experiment, seedlings were established in seedling trays for 20 days before transplanting to pots. Pots treated with control—S0 (no salt—0.4 ds/m) and saline—S1 (6.84 ds/m) in the previous study were treated with respective salt levels to maintain 6.84 ds/m EC in respective pots. In the current study, saline irrigation water was applied in respective pots to maintain the required salinity level (6.84 ds/m EC) in paddy conditions. Plants in non-salinity treatment groups received tap water with no added salt throughout the experiment until harvest. A salinometer (WS-200 PLUS, Dongguan Shengshan electronic Technology Ltd, Dongguan, China) utilizing time domain technology was used to monitor salinity levels in the treatment pots. To avoid including the reproductive stage, all plants were harvested 70 days after sowing. Prior to harvest, several plant growth parameters were measured for both rice varieties: plant height, number of tillers, and total aboveground biomass. The fresh weight of the plants (g/pot) was recorded at harvest. Subsequently, the fresh biomass samples were oven-dried at 105 °C for 48 h to determine their dry weight. The post-harvest soil was analyzed for changes in chemical properties during the experiment; the details are presented in Supplementary Information Section S1.

### 4.2. Physiological Performance of Rice Plants

Chlorophyll content in leaves was measured using a SPAD meter (SPAD-502, Minolta Co. Ltd., Tokyo, Japan) on one randomly selected plant per replicate. Three SPAD readings were taken from fully mature leaves, starting from the top of the plant. Photosynthetic performance was further evaluated for selected treatments using a portable DUAL-PAM-100: P700 (Effeltrich, Germany) & Chlorophyll Fluorescence system. This instrument measured various parameters, including chlorophyll fluorescence (Fv/Fm), non-photochemical quenching (NPQ), photochemical quenching coefficient (qP), and photon yield of Photosystem II (ΦPSII).

### 4.3. Biochemical Analysis of Rice Plant Leaves

Following the method of Guo et al. [22], fresh leaf samples from each treatment were homogenized in a pre-chilled mortar and pestle with 0.05 M sodium phosphate buffer (pH 7.8) after each 5-day interval. The homogenate was then centrifuged at 10,000× *g* for 20 min at 4 °C. The resulting supernatant was used for subsequent analyses of superoxide dismutase (SOD), peroxidase (POD), and catalase (CAT) activity. Activities of malondialdehyde (MDA), superoxide dismutase (SOD), peroxidase (POD), and catalase (CAT) were determined using a modification of the method described by [84]. The relative water content (RWC) and membrane stability index (MSI) of rice leaves for both varieties were measured; the details are presented in Supplementary Information Section S2.

### 4.4. Measurement of Na^+^ and K^+^ Content in Leaves

Prior to the analysis of leaf sodium (Na^+^) and potassium (K^+^) content, leaf samples were meticulously washed with deionized water (ddH_2_O) to eliminate any surface-adhered salt particles. Following the wash, the leaves were gently patted dry with tissue paper. To ensure complete moisture removal, they were then oven-dried at 80 °C until reaching a constant weight. Finally, the dried leaves were ground into a fine powder using a mechanical grinder (Retsch MM 400, Haan, Germany). For analysis of leaf Na^+^ and K^+^ content, 0.1 g of ground sample was weighed into a 50 mL glass tube. To this, 0.2 mL of deionized water (ddH_2_O) and 5 mL of concentrated sulfuric acid (H_2_SO_4_) were added. The samples were then placed in a digestion instrument (LWY84B, Siping Institute of Electronics Technique, Siping, China) for further processing. After incubation for 1.5 h, a small amount (0.2 mL) of 30% hydrogen peroxide (H_2_O_2_) was added to each sample. The tubes were mixed for 30 min and then returned to the digestion instrument until white fumes appeared, indicating complete digestion of the organic matter. Upon observing white fumes, signifying complete sample digestion, the tubes were cautiously removed from the digestion instrument and allowed to cool. Subsequently, deionized water (ddH_2_O) was added to each tube, bringing the final volume to 50 mL. For Na^+^ and K^+^ determination, a flame photometer (FP 640, Shanghai INESA Scientific Instrument Co., Ltd. China) was calibrated using a standard curve following the manufacturer’s instructions. The corresponding Na^+^ and K^+^ concentrations in the samples were then calculated using the method described by Khan et al. [85].

### 4.5. Scanning Electron Microscopy (SEM) of Root Cross Section and Transmission Electron Microscopy (TEM) of Leaf Cells

Three small root cross sections (1 mm^2^) were excised from the middle portion of roots for each selected treatment. These samples were rinsed with distilled water to remove any surface debris before electron microscopy analysis. To preserve the samples for observation, they were subsequently fixed in a solution of 4% glutaraldehyde and 0.2 M sodium phosphate buffer (pH 6.8) for 6 h at 4 °C. Following fixation, the samples underwent four washes with 0.1 M sodium phosphate buffer (pH 6.8) to remove fixative residues. Subsequently, they were dehydrated through a graded series of ethanol solutions. After dehydration, the samples were rinsed twice with isoamyl acetate and then freeze-dried. Small sections of the freeze-dried root samples were securely mounted onto specimen stubs using double-sided tape. Finally, a thin layer of gold was sputtered onto the samples using an Ion Sputter Coater (J20, Beijing Technol Science Co., Ltd. China) for improved conductivity during electron microscopy analysis [21,74]. Finally, the images of the root cross-section were examined using a scanning electron microscope (Thermo Scientific Verios, G4 UC, Thermo Fisher Scientific Inc. Waltham, Massachusetts, USA). Image J software, V.1.53t) was then employed to determine the total area of the root cross-section (TA), vascular cylinder (VC), and cortical parenchyma (CP) of the root cross-section. For transmission electron microscopy (TEM) analysis, samples underwent post-fixation in a solution of 1% osmic acid buffered with 0.2 M sodium phosphate (pH 6.8). Dehydration was achieved through a graded ethanol series, followed by critical point drying. To enhance contrast for TEM observation, the thin leaf sections were stained with lead citrate and 2% uranyl acetate. Finally, the samples were visualized using a Hitachi 500 electron microscope (Hitachi High-Tech Corporation, Tokyo, Japan) [74].

### 4.6. Statistical Analysis

Statistical analyses were performed using the software package Statistics 8.1. Data are presented as means ± standard deviation. To assess treatment effects, a three-way analysis of variance (ANOVA) was conducted, followed by a least significant difference (LSD) test at a significance level of *p* < 0.05.

## 5. Conclusions

Salt stress negatively affects the chemical properties of soil and the biochemical, physiological, anatomical, and ultimately morphological attributes of rice plants. While residual effects of microbial-inoculated biochar (BB) with N fertilizer reduced salt accumulation in the soil as well as in plants by improving soil properties and biochemical and physiological attributes of plants ultimately enhancing plant growth. Best rice growth was observed under BB + N120 treatment in saline conditions, and SLY138 showed significantly more resistance to salt stress than JLY534 when irrigated with saline water. Thus, the application of bacterial inoculated biochar with N fertilizer as a long-term mitigation strategy for rice growth successfully would be a better strategy under saline conditions. Further, microbial-inoculated biochar combined with other inorganic fertilizers should be evaluated as a remediation strategy for saline water and soil. It would also be useful to investigate possible long-term adverse effects and impacts on crop genetic diversity.

## Figures and Tables

**Table 1:** Effect of microbial-inoculated biochar and N on morphology and SPAD values of rice plants under saline conditions.

		Plant Height (cm)		Plant Fresh Weight (g/Pot)		Plant Dry Weight (g/Pot)		No. of Tillers/Pot		SPAD	
Treatment	EC (ds/m)	SLY138	JLY534	SLY138	JLY534	SLY138	JLY534	SLY138	JLY534	SLY138	JLY534
N60	0.4	32.58 t	28.85 tu	37.75 vw	33.33 wx	16.35 s–u	15.35 t–v	13.00 u–x	8.25 yz	25.63 n–q	23.63 p–s
	6.84	23.50 v	17.53 w	22.45 yz	17.03 z	10.23 wx	4.10 y	7.75 y–z	5.00 z	19.20 r–t	14.08 t
N120	0.4	49.48 m–o	46.08 n–q	53.38 rs	49.18 st	22.95 n–q	19.00 q–t	20.00 o–s	16.50 r–v	26.80 m–q	24.65 q–r
	6.84	38.18 rs	33.85 st	37.15 vw	28.08 xy	14.33 u–w	6.70 xy	12.25 v–y	10.75 w–y	21.43 q–s	18.43 st
BC	0.4	41.23 qr	38.83 rs	45.40 tu	41.25 uv	20.38 q–s	18.58 r–t	17.00 r–u	14.00 t–w	33.83 e–l	33.23 f–l
	6.84	33.90 st	27.03 uv	28.63 xy	24.43 y	12.80 u–w	6.38 x–y	9.75 w–y	9.25 x–z	30.05 k–o	28.13 l–p
BF	0.4	61.08 ij	59.03 jk	114.90 j	111.55 jk	29.93 k	29.00 i–l	25.50 i–m	22.75 l–q	37.30 b–i	34.68 d–k
	6.84	49.78 mn	47.28 n–p	95.45 no	89.23 op	21.65 o–r	13.15 u–w	18.25 q–t	16.00 s–v	34.73 d–k	32.90 g–l
BB	0.4	73.55 ef	71.93 e–g	129.75 hi	125.30 i	35.05 e–g	33.63 f–h	29.25 f–i	28.00 g–j	38.80 b–g	36.13 c–j
	6.84	60.90 i–k	56.23 j–l	107.75 kl	104.05 lm	25.45 l–o	16.95 s–u	23.00 k–p	19.00 p–s	37.20 b–i	31.80 i–m
BC + N60	0.4	55.78 kl	53.70 lm	78.40 q	74.63 q	29.20 i–l	26.55 k–n	24.25 j–o	21.75 m–q	36.25 c–j	34.28 d–k
	6.84	44.35 o–q	42.25 p–r	55.68 r	42.33 tu	20.58 p–s	11.50 vw	17.00 r–u	16.25 r–v	35.78 c–k	30.73 j–n
BC + N120	0.4	68.48 f–h	67.45 f–h	105.05 lm	100.63 mn	34.58 e–g	32.08 g–i	28.00 g–j	25.25 i–n	38.88 b–g	35.98 c–k
	6.84	55.85 j–l	52.95 lm	87.25 p	77.08 q	24.75 m–p	15.65 t–v	20.75 n–r	19.25 p–s	34.90 c–k	32.48 h–m
BF + N60	0.4	82.30 c	80.35 c	146.40 de	139.85 f	42.58 cd	38.70 de	33.50 c–f	32.00 d–g	39.25 b–f	38.48 b–h
	6.84	71.83 e–g	65.45 hi	125.55 i	116.83 j	31.40 g–j	21.60 o–r	27.00 h–l	23.50 j–p	35.35 c–k	35.33 c–k
BF + N120	0.4	91.65 b	89.65 b	157.13 bc	153.65 c	46.45 bc	43.95 c	36.50 b–d	35.50 b–e	40.85 bc	39.75 b–e
	6.84	78.85 cd	71.25 e–g	136.50 fg	128.73 hi	35.18 e–g	27.28 j–m	30.25 f–h	27.00 h–l	37.70 b–i	34.90 c–k
BB + N60	0.4	93.15 b	92.10 b	161.65 b	156.78 bc	48.60 b	45.50 bc	38.25 b	37.00 bc	40.18 b–d	36.83 c–i
	6.84	83.38 c	74.23 de	140.55 ef	131.98 gh	37.15 ef	30.10 h–k	31.50 e–h	27.50 g–k	36.98 b–i	34.18 d–k
BB + N120	0.4	106.25 a	102.50 a	176.58 a	175.35 a	55.30 a	53.68 a	43.25 a	44.25 a	46.98 a	42.88 ab
	6.84	92.68 b	81.65 c	152.45 cd	146.53 de	43.93 c	36.43 ef	36.00 b–e	30.50 f–h	37.95 b–h	34.63 d–k
S		*		*		*		*		*	
T		*		*		*		*		*	
V		*		*		*		*		*	
T × S × V		ns		ns		ns		ns		ns	

The means that have the same letter do not differ substantially at *p* > 0.05 for a parameter, significant * (*p* ≤ 0.05), non-significant (ns; *p* > 0.05). T (Treatment), S (Salt), V (Variety).

**Table 2:** Effect of microbial-inoculated biochar and N (selective treatments) on agronomic attributes and SPAD of rice plants under saline conditions.

		Plant Height (cm)		Plant Fresh Weight (g/Pot)		Plant Dry Weight (g/Pot)		No. of Tillers/Pot		SPAD	
Treatment	EC (ds/m)	SLY138	JLY534	SLY138	JLY534	SLY138	JLY534	SLY138	JLY534	SLY138	JLY534
BC	0.4	41.23 h	38.83 hi	45.40 j	41.25 j	20.38 g	18.58 g	17.00 h	14.00 hi	33.83 d–h	33.23 e–h
	6.84	33.90 i	27.03 j	28.63 k	24.43 k	12.80 h	6.38 i	9.75 i	9.25 i	30.05 gh	28.13 h
BF	0.4	61.08 f	59.03 f	114.90 f	111.55 fg	29.93 de	29.00 de	25.50 d–f	22.75 fg	37.30 b–f	34.68 c–g
	6.84	49.78 g	47.28 g	95.45 i	89.23 i	21.65 fg	13.15 h	18.25 gh	16.00 h	34.73 c–g	32.90 e–h
BB	0.4	73.55 de	71.93 e	129.75 de	125.30 e	35.05 c	33.63 cd	29.25 d	28.00 de	38.80 b–e	36.13 c–g
	6.84	60.90 f	56.23 f	107.75 gh	104.05 h	25.45 ef	16.95 gh	23.00 e–g	19.00 gh	37.20 b–f	31.80 f–h
BF + N120	0.4	91.65 b	89.65 b	157.13 b	153.65 b	46.45 b	43.95 b	36.50 b	35.50 bc	40.85 a–c	39.75 b–d
	6.84	78.85 cd	71.25 e	136.50 d	128.73 e	35.18 c	27.28 e	30.25 d	27.00 d–f	37.70 b–f	34.90 c–g
BB + N120	0.4	106.25 a	102.50 a	176.58 a	175.35 a	55.30 a	53.68 a	43.25 a	44.25 a	46.98 a	42.88 ab
	6.84	92.68 b	81.65 c	152.45 bc	146.53 c	43.93 b	36.43 c	36.00 b	30.50 cd	37.95 b–f	34.63 c–h
S		*		*		*		*		*	
T		*		*		*		*		*	
V		*		*		*		*		*	
T × S × V		ns		ns		ns		ns		ns	

Parameters share the same alphabets indicating that there is a non-significant difference when the *p*-value is greater than or equal to 0.05. ns; non-significant, *; significant (*p* ≤ 0.05). T (treatment); S (salt); V (variety).

## Data Availability

Data will be made available on request.

## Supplementary Materials

The following supporting information can be downloaded at: https://www.mdpi.com/article/10.3390/plants13192804/s1, The supplementary file includes Section S1. Chemical properties of post-harvest soil (References [86,87,88]); Section S2. Relative water content and membrane stability index (References [89,90]); Table S1. Treatment plan for experiment; Table S2. Selected treatments on the base of best performance in morphology of rice plants; Table S3. Soil properties prior to first growing season (native soil) and residual growing season for SLY138; Table S4. Soil properties prior to the first growing season (native soil) and residual growing season for JLY534.
