# Supplementary material for: Residual Effect of Microbial-Inoculated Biochar with Nitrogen on Rice Growth and Salinity Reduction in Paddy Soil
Source: Plants (Basel). 2024 Oct 6;13(19):2804. doi: 10.3390/plants13192804 (PMC11478880; doi:10.3390/plants13192804)
Supplement: Supplementary file 1 [file plants-13-02804-s001.zip › plants-3217813-supplementary.pdf]

Residual Effect of Microbial-Inoculated Biochar with Nitrogen on Rice Growth and Salinity Reduction in Paddy Soil

Hafiz Muhammad Mazhar Abbas [1], Ummah Rais [2], Haider Sultan [1], Ashar Tahir [3], Saraj Bahadur [4], Asad Shah [1], Asim Iqbal [5], Yusheng Li [1], Mohammad Nauman Khan [1,*] and Lixiao Nie [1,*]

[1] School of Breeding and Multiplication (Sanya Institute of Breeding and Multiplication), Hainan University, Sanya 572000, China; mazharabbas@hainanu.edu.cn (H.M.M.A.); sultanhaider@hainanu.edu.cn (H.S.); 184459@hainanu.edu.cn (A.S.); yushengli@hainanu.edu.cn (Y.L.)
[2] Department of Zoology, The Islamiyah University of Bahawalpur, Bahawalpur 63100, Pakistan; ummahasghar2301@gmail.com
[3] School of Tropical Agriculture and Forestry, Hainan University, Haikou 570228, China; drashartahir@hainanu.edu.cn
[4] College of Forestry, Hainan University, Haikou 570228, China; saraj@hainanu.edu.cn
[5] Department of Agronomy, University of Agriculture Faisalabad, Faisalabad 37000, Pakistan; aximiqbal60@gmail.com
* Correspondence: 184268@hainanu.edu.cn (M.N.K.); lxnie@hainanu.edu.cn (L.N.)

This file include 2 Sections, and 4 tables

Section S1: Chemical properties of post-harvest soil

Rhizosphere soil samples were collected manually from selected treatments. After air-drying, the samples were meticulously cleaned to remove stones and any remaining plant material (residual litter). The dried and cleaned soil was then ground to a fine powder using a 2-millimeter sieve. Finally, the prepared soil was stored for subsequent analyses. A subsample of the air-dried soil (< 2 mm) was further ground to an even finer fraction that could pass through a 0.15-millimeter sieve. This finer fraction was then used for subsequent soil analyses, including the determination of organic matter content using the [1,2]. Soil pH was determined using a 1:2 soil-to-water ratio and a pH meter. Exchangeable sodium (Na^+) and potassium (K^+) content were measured using a separate extraction and analysis method [3].

Exchangeable Na (mg/ kg) = Extractable Na (mg/kg) - Soluble Na (mg/kg)

Exchangeable K (mg/ kg) = Extractable K (mg/kg) – Soluble K (mg/kg)

Extractable and soluble Na^+ and K^+ were measure in accordance with phlame photmeter. Ammonium nitrogen (NH_4^+-N) and nitrate nitrogen (NO_3^--N) were quantified using the indophenol blue method [3].

Section S2: Relative water content and membrane stability index

Leaf relative water content (RWC) was determined using the gravimetric method [4]. Fresh leaf samples (0.5 g) were weighed (HR-60 precision balance) and then submerged in water for 4 hours to achieve turgor. Subsequently, the leaves were oven-dried at high temperature until a constant weight was reached. The RWC was calculated using the formula provided below.

$$RWC (\%) = [((FW-DW))/((TW-DW)] \times 100)$$

The membrane stability index (MSI) was estimated following the protocol described by [5]. Briefly, 50 mg of leaf material was weighed out in duplicate and placed in test tubes containing 10 mL of double-distilled water. One set of samples was incubated in a water bath at 40°C for 30 minutes (C1). The electrical conductivity of the solution was then measured using a conductivity meter (CL-250) on a conductivity bridge. The second set of samples was boiled in a water bath for 10 minutes (C2), and their conductivity was also measured using the conductivity bridge. The MSI was calculated using the formula provided below,

$$MSI (\%) = [1- (C1/C2)] \times 100$$

Table S1: Treatment plan for experiment

Treatments	salt levels	Treatment Name	Application Rates
T1	0%	N60	Nitrogen @ 60 kg ha^{-1}
	0.40%	N60	Nitrogen @ 60 kg ha^{-1}
T2	0%	N120	Nitrogen @ 120 kg ha^{-1}
	0.40%	N120	Nitrogen @ 120 kg ha^{-1}
T3	0%	BC	Simple Rice Straw biochar (1%)
	0.40%	BC	Simple Rice Straw biochar (1%)
T4	0%	BF	Fungal Inoculated biochar (1%)
	0.40%	BF	Fungal Inoculated biochar (1%)
T5	0%	BB	Bacterial Inoculated biochar (1%)
	0.40%	BB	Bacterial Inoculated biochar (1%)
T6	0%	BC+N60	Simple Rice Straw biochar (1%) + Nitrogen @ 60 kg ha^{-1}
	0.40%	BC+N60	Simple Rice Straw biochar (1%) + Nitrogen @ 60 kg ha^{-1}
T7	0%	BC+N120	Simple Rice Straw biochar (1%) + Nitrogen @ 120 kg ha^{-1}
	0.40%	BC+N120	Simple Rice Straw biochar (1%) + Nitrogen @ 120 kg ha^{-1}
T8	0%	BF+N60	Fungal Inoculated biochar (1%) + Nitrogen @ 60 kg ha^{-1}
	0.40%	BF+N60	Fungal Inoculated biochar (1%) + Nitrogen @ 60 kg ha^{-1}
T9	0%	BF+N120	Fungal Inoculated biochar (1%) + Nitrogen @ 120 kg ha^{-1}
	0.40%	BF+N120	Fungal Inoculated biochar (1%) + Nitrogen @ 120 kg ha^{-1}
T10	0%	BB+N60	Bacterial Inoculated biochar (1%) + Nitrogen @ 60 kg ha^{-1}
	0.40%	BB+N60	Bacterial Inoculated biochar (1%) + Nitrogen @ 60 kg ha^{-1}
T11	0%	BB+N120	Bacterial Inoculated biochar (1%) + Nitrogen @ 120 kg ha^{-1}
	0.40%	BB+N120	Bacterial Inoculated biochar (1%) + Nitrogen @ 120 kg ha^{-1}

Table S2: Selected treatments on the base of best performance in morphology of rice plants

Treatments	salt levels	Treatment Name	Application Rates
T3	0%	BC	Simple Rice Straw biochar (1%)
	0.40%	BC	Simple Rice Straw biochar (1%)
T4	0%	BF	Fungal Inoculated biochar (1%)
	0.40%	BF	Fungal Inoculated biochar (1%)
T5	0%	BB	Bacterial Inoculated biochar (1%)
	0.40%	BB	Bacterial Inoculated biochar (1%)
T9	0%	BF+N120	Fungal Inoculated biochar (1%)+Nitrogen @ 120 kg ha^{-1}
	0.40%	BF+N120	Fungal Inoculated biochar (1%)+Nitrogen @ 120 kg ha^{-1}
T11	0%	BB+N120	Bacterial Inoculated biochar (1%) + Nitrogen @ 120 kg ha^{-1}
	0.40%	BB+N120	Bacterial Inoculated biochar (1%) + Nitrogen @ 120 kg ha^{-1}

Table S3: Soil properties prior to first growing season (native soil) and residual growing season for SLY138

Attributes	prior to 1st experiment	Non-saline					Saline				
		BC	BF	BB	BF+N	BB+N	BC	BF	BB	BF+N	BB+N
pH	6.51 b	7.5 a	6.29 b	6.08 b	5.929 bc	5.54 c	8.67 a	7.62 b	7.32 b	6.99 bc	6.65 c
OM %	0.105 c	1.5 b	1.56 b	1.88 ab	1.91 ab	2.08 a	1.11 c	1.19 c	1.32 bc	1.46 b	2.77 a
NO_3^--N (mg kg^{-1})	0.95 e	1.5 d	2.25 c	2.88 b	3.91 ab	4.17 a	1.12 c	1.11 c	1.21 c	3.02 b	3.51 a
NH_4^+-N (mg kg^{-1})	1.87 e	2.5 cd	3.22 bc	3.59 b	4.46 ab	4.93 a	2.32 e	3.15 d	3.55 c	4.42 b	4.96 a
Exchangeable Na^+ (mg kg^{-1})	-	16.13 ab	17.12 a	17.38 a	16.26 ab	16.90 ab	59.52 a	50.10 b	44.02 c	40.02 d	33.13 e
Exchangeable K^+ (mg kg^{-1})	-	30.34 cd	31.79 cd	35.3 c	45.08 b	62.37 a	13.83 e	21.38 d	26.50 c	31.03 b	51.58 a

Table S4: Soil properties prior to first growing season (native soil) and residual growing season for JLY534

Attributes	prior to 1st experiment	Non-saline					Saline				
		BC	BF	BB	BF+N	BB+N	BC	BF	BB	BF+N	BB+N
pH	6.51 b	7.55 a	6.42 b	6.29 bc	5.97 c	5.45 d	8.69 a	7.65 b	7.27 bc	6.95 c	6.67 cd
OM %	0.105 d	1.44 c	1.69 b	1.74 b	1.89 ab	2.08 a	1.15 bc	1.19 bc	1.34 abc	1.54 ab	1.61 a
NO_3^--N (mg kg^{-1})	0.95 e	1.39 d	2.21 c	2.71 b	3.08 ab	3.34 a	0.99 e	1.02 d	1.13 c	2.43 b	2.84 a
NH_4^+-N (mg kg^{-1})	1.87 e	2.03 d	2.93 c	3.49 b	4.45 a	4.64 a	1.94 d	2.79 c	3.2 b	4.35 ab	4.56 a
Exchangeable Na^+ (mg kg^{-1})	-	19.42 a	18.90 ab	20.07 a	19.41 a	20.09 a	58.11 a	49.46 b	43.56 c	34.66 d	32.76 de
Exchangeable K^+ (mg kg^{-1})	-	25.98 e	30.35 d	36.56 c	43.87 b	60.05 a	9.35 e	19.32 d	23.30 c	27.48 b	45.14 a

References

1. FAO The Euphrates Pilot Irrigation Project. Methods of Soil Analysis. Gadeb Soil Laboratory (a Laboratory Manual) 1974.

2. Walkley, A. A Critical Examination of a Rapid Method for Determining Organic Carbon in Soils—Effect of Variations in Digestion Conditions and of Inorganic Soil Constituents. *Soil Sci.* **1947**, *63*, 251–264.

3. Maynard, D.G.; Kalra, Y.P.; Crumbaugh, J.A. Nitrate and Exchangeable Ammonium Nitrogen. *Soil Sampl. methods Anal.* **1993**, *1*, 25–38.

4. Weatherley, P. Studies in the Water Relations of the Cotton Plant. I. The Field Measurement of Water Deficits in Leaves. *New Phytol.* **1950**, 81–97.

5. Sairam, R.K.; Deshmukh, P.S.; Shukla, D.S. Tolerance of Drought and Temperature Stress in Relation to Increased Antioxidant Enzyme Activity in Wheat. *J. Agron. Crop Sci.* **1997**, *178*, 171–178.
